# Genomic Signatures of Freshwater Adaptation in Pacific Herring (*Clupea pallasii*)

**DOI:** 10.3390/genes13101856

**Published:** 2022-10-14

**Authors:** Artem Nedoluzhko, Svetlana Yu. Orlova, Denis S. Kurnosov, Alexei M. Orlov, Jorge Galindo-Villegas, Sergey M. Rastorguev

**Affiliations:** 1 Paleogenomics Laboratory, European University at Saint Petersburg, 191187 Saint Petersburg, Russia; 2 Limited Liability Company ELGENE, 109029 Moscow, Russia; 3 Laboratory of Molecular Genetics, Russian Federal Research Institute of Fisheries and Oceanography, 107140 Moscow, Russia; 4 Laboratory of Genetic Basis of Identification, Vavilov Institute of General Genetics of the Russian Academy of Sciences, 119991 Moscow, Russia; 5 Research Group of Intraspecific Differentiation, Russian Federal Research Institute of Fisheries and Oceanography, Pacific Branch (TINRO), 690091 Vladivostok, Russia; 6 Laboratory of Oceanic Ichthyofauna, Shirshov Institute of Oceanology of the Russian Academy of Sciences, 117218 Moscow, Russia; 7 Laboratory of Behavior of Lower Vertebrates, A.N. Severtsov Institute of Ecology and Evolution, Russian Academy of Sciences, 119071 Moscow, Russia; 8 Department of Ichthyology, Dagestan State University, 367000 Makhachkala, Russia; 9 Department of Ichthyology and Hydrobiology, Tomsk State University, 634050 Tomsk, Russia; 10 Laboratory of Marine Biology, Caspian Institute of Biological Resources, Russian Academy of Sciences, 367000 Makhachkala, Russia; 11 Genomics Division, Faculty of Biosciences and Aquaculture, Nord University, 8049 Bodø, Norway; 12 Kurchatov Center for Genomic Research, National Research Centre “Kurchatov Institute”, 123182 Moscow, Russia

**Keywords:** speciation, subspecies, ecological form, RAD sequencing, marine, lake, isolation, Russia

## Abstract

Pacific herring (*Clupea pallasii*) is an essential target of commercial fishing in the North Pacific Ocean. Previous studies have suggested the existence of marine and lake ecological forms of this species within its range. The lake ecological form of herring has a shortened life cycle, spending the winter and spawning in brackish waters near the shoreline without long migrations for feeding; it also has a relatively smaller body size than the marine form. Genetic-based studies have shown that brackish water Pacific herring not only can be distinguished as a separate lake ecological form but possibly has its genetic legacy. Here, as part of an ongoing study, using ddRAD-sequencing data for marine and lake ecological forms from a total of 54 individuals and methods of comparative bioinformatics, we describe genomic signatures of freshwater adaptivity in Pacific herring. In total, 253 genes containing discriminating SNPs were found, and part of those genes was organized into genome clusters, also known as “genomic islands of divergence”. Moreover, the Tajima’s D test showed that these loci are under directional selection in the lake populations of the Pacific herring. Yet, most discriminating loci between the lake and marine ecological forms of Pacific herring do not intersect (by gene name) with those in other known marine fish species with known freshwater/brackish populations. However, some are associated with the same physiological trait—osmoregulation.

## 1. Introduction

Freshwater adaptation among marine fish species opens them new ecological niches and evolutionary opportunities. In contrast, this environmental shift requires substantial changes in immunity, physiological and metabolic processes, osmoregulation, and behavior [1]. The genetic basis of adaptation to different salinity environments has been studied for the different teleost species—prickly sculpin (*Cottus asper*) [2], mummichog (*Fundulus heteroclitus*) [3], Atlantic cod (*Gadus morhua*) [4], rainwater killifish (*Lucania parva*) [5]. Genetic comparisons between anadromous and nonmigratory ecotypes of rainbow trout (*Oncorhynchus mykiss*) and Atlantic salmon (*Salmo salar*) were also carried out [1,6,7].

Significant advances have been achieved during in-depth studies of freshwater adaptation of the three-spined stickleback (*Gasterosteus aculeatus*), the well-established model for modern evolutionary biology [8]. Genetic comparisons in this species have been intensively studied in the migratory and nonmigratory ecotypes. Such studies are based on genomic data [9,10,11,12,13] and gene expression of protein-coding genes [14] or microRNAs [15]. These studies showed that the genes responsible for ion transmembrane transport play an essential role in adaptation to the freshwater environment. Moreover, these genes are under positive selection [16]. In many cases, genomic regions responsible for adaptation to the freshwater environments are grouped into compact genomic islands of divergence [4,10,11]. Atlantic herring (*Clupea harengus*) also has interpopulation genetic differentiation, which is related to different salinity levels and is associated with haplotype blocks, often spanning multiple genes and maintained by balancing selection [17].

The commercial importance of the Pacific herring (*C. pallasii*) and its trophic significance for other species in marine ecosystems prompt many studies aimed at its biology, reproduction, diversity, and genetic differentiation [18,19,20,21,22,23,24]. This species has marine and lake ecological forms. The marine ecological form of *C. pallasii* performs long feeding migrations and spends wintertime in the upper part of the deep sea. In addition, it uses to spawn in large sea bays. Conversely, the lake ecological form prefers brackish lagoon-type lakes and small bays to spawn and spend the winter [25]. Recent genetic-based studies showed the level of genetic differentiation between populations from the Russian part of the North Pacific Ocean and the Kara Sea; moreover, lake populations from the Kamchatka Peninsula and Sakhalin Island separated from the marine populations of Pacific herring [21,26]. At the same time, it has been suggested that the lake ecological form has its genetic component associated with freshwater adaptation [26].

In this study, we present the analysis of a double digestion restriction site-associated DNA (ddRAD) sequencing dataset of 54 individuals of Pacific herring, which was previously determined as lake (15 specimens) and marine (39 specimens) ecological forms. Genotype analysis of marine and lake ecological forms allows us to identify the genomic signatures of freshwater adaptation in Pacific herring and carry out the comparative analysis of freshwater adaptation loci with its congener—Atlantic herring.

The comparison of such discriminating loci in the genomes of marine fish species with known freshwater adaptation ability will determine the genetic mechanisms and directions of adaptation to the different osmotic conditions, particularly during the conquering of new freshwater ecological niches. It is assumed that other environmental factors related to the new habitat are also changing in addition to osmotic conditions. Another critical topic considered in this study is the representation of new data on how rigidly determined the evolutionary mechanisms of adaptation to an environmental condition and the habitats of different species. From a general point of view, it is assumed that such mechanisms may differ significantly in evolutionarily distant species in comparison with close evolutionary species [27]. Nevertheless, it involves common genes or gene categories that underlie adaptive traits. The comparative analysis of such adaptation-associated loci sheds light on the species-specific genomic mechanisms in higher vertebrates.

## 2. Materials and Methods

### 2.1. Pacific Herring Genomic Dataset Description

**Figure 1:**
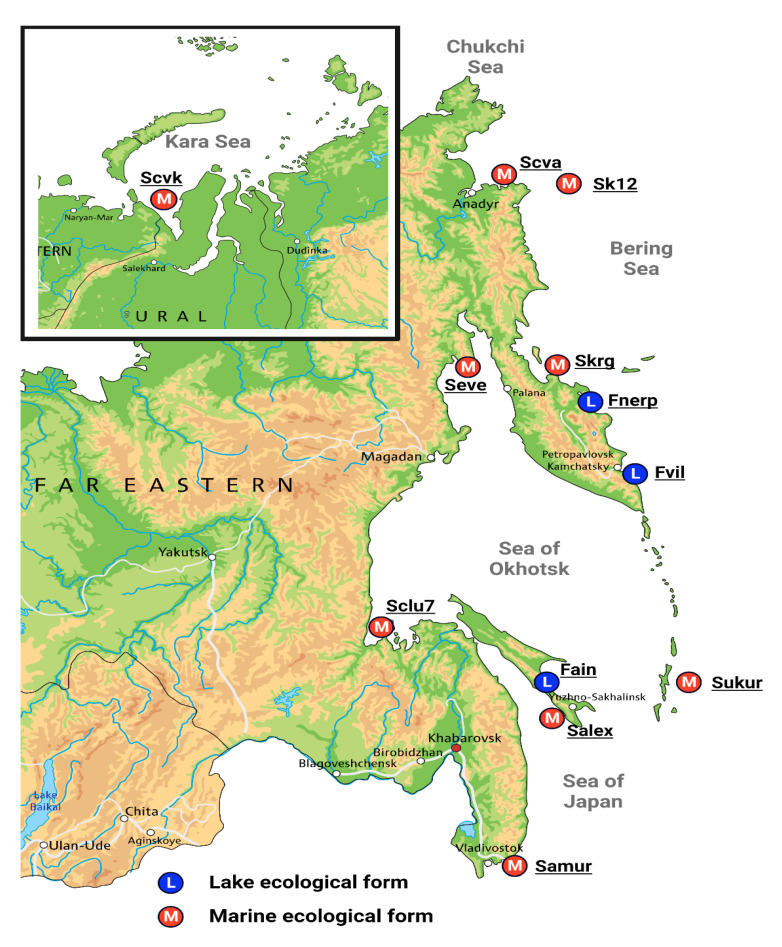
Map showing the sampling sites of lake (marked by blue circles) and marine (marked by red circles) Pacific herring (*Clupea pallasii*) populations. The Pacific herring specimen description is presented in Table 1.

The sampling, DNA extraction, ddRAD library preparation and DNA sequencing were previously described [26]. Fifty-four individuals of two ecological forms of Pacific herring from the Northwest Pacific and the Kara Sea were involved in the present study. Specimens from twelve wild populations of Pacific herring were combined as two separate marine (39 specimens) and lake (15 specimens) ecological forms and used in a comparative analysis aimed at describing genomic signatures of freshwater adaptation (Table 1; Figure 1). The average salinity and temperature in the sampling regions in late spring—early summer are presented in Table 2.

### 2.2. Bioinformatics Analysis

Raw DNA reads were converted to FASTQ format and demultiplexed using bcl2fastq (v2.20). DNA reads quality was examined with the FastQC tool (v0.11.5) [38]. Library adapter trimming from the sequencing data and quality filtration (phred 30) were carried out using cutadapt (v2.10) [39].

Stacks package (v2.41) and its clone_filter module were used for PCR duplicate removal. In addition, the process_radtags module from the same package was used for dual index demultiplexing and additional quality filtration [40]. The output reads from the Stacks package were mapped against the Atlantic herring reference genome (Ch_v2.0.2, https://www.ncbi.nlm.nih.gov/assembly/GCF_900700415.2 (accessed on 4 July 2022)) using Bowtie2 under the “very-sensitive” parameter set [41]. The BAM files that were obtained using the Samtools (v1.7) [42] and uploaded to BCFtools (v1.9) [42] for SNP-calling. Only SNPs with coverage higher than 1000× for all 54 Pacific herring individuals were used in the subsequent analyses.

The filtered VCF file was loaded into the R statistical environment (v3.4.4) for discriminant analysis. The VCF file was then converted into genlight format and used for discriminant analysis of principal components (DAPC) in the adegenet R package (v2.1.3) [43].

The VCF file was also converted into internal plink format using PLINK (v1.9) package under the “make-bed” parameter [44]. To find the loci in which allele frequencies differ in marine and lake populations of Pacific herring, we used loci association analysis using the “assoc” command, as in the case–control association study, with Fisher’s *p*-value determination. Loci with *p*-value less than 1 × 10^−6^ were considered to discriminate between marine and lake Pacific herring ecological forms. Gene names with discriminating SNP loci were determined using coordinates of genes from the Atlantic herring reference genome (Ch_v2.0.2), SNPs coordinates, and bedtools software package (v2.27.1) under the “intersect” command [45].

The gene list, containing discriminating SNP loci was analyzed against the reference group of genes using the ShinyGO (v0.76) web service [46]. The genes with a single discriminating SNP in the filtered VCF dataset were also included in the comparison. A sliding window method from the ShinyGO tool was implicated in the genomic region prediction process with the SNPs that discriminate marine and lake ecological forms of Pacific herring. The window size was equal to two megabase pairs (Mbp). The hypergeometric test was used to determine if the genes were significantly overrepresented. The FDR cutoff for each window was equal to 0.001.

The neutrality test was performed by Tajima’s D method [47] using the Tajima command of the VCF-kit package [48]. The test was performed by evaluating the Tajima value in sliding windows with a length of 10,000 bp. The distribution of the Tajima D test values for frames containing discriminating genes was compared with those containing the remaining genes using the t.test function of the R environment. Analyses were carried out separately for the VCF file, which contained all Pacific herring specimen SNPs data, and for a VCF file which had only lake Pacific herring specimen SNPs data.

## 3. Results

### 3.1. Marine and Lake Ecological Form of Pacific Herring Dataset Description and Mapping Statistics

The total number of reads generated for 54 lake and marine specimens of Pacific herring was 216,433,546 (NCBI accession numbers are presented in Table 1). DNA reads were mapped to the reference genome of Atlantic herring (Ch_v2.0.2, https://www.ncbi.nlm.nih.gov/assembly/GCF_900700415.2 (accessed on 4 July 2022)) after adapter trimming and quality filtration. From 59.91 to 88.48% of reads per ddRAD library were mapped to the reference (Table S1). Only SNPs with coverage higher than 1000× (*p < 0.05*) were used after the SNPs calling.

### 3.2. Distribution of Discriminating Loci between Marine and Lake Ecological Forms of Pacific Herring

**Figure 2:**
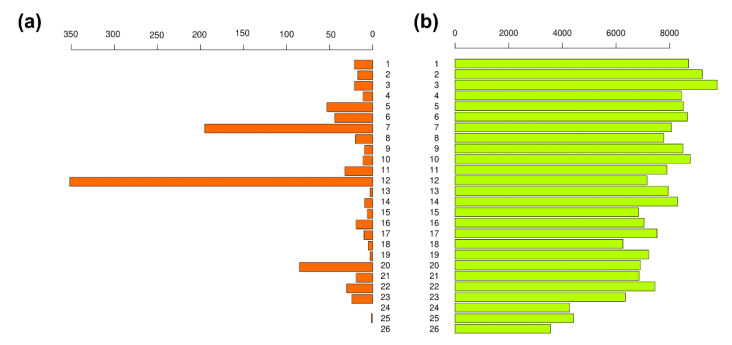
Bar plots represent the number of SNP loci identified for Pacific herring’s marine and lake ecological forms. (**a**) Barplot shows the number of discriminating loci for Pacific herring’s marine and lake ecological forms. (**b**) The barplot shows the total number of SNP loci for each chromosome. The *X*-axis represents the number of SNP loci. The *Y*-axis represents chromosome id.

A total of 192,433 SNP loci from the 26 chromosomes of the Atlantic herring genome were involved in the PLINK analysis. The coverage of chromosomes by DNA reads was approximately uniform, and the number of SNP loci per chromosome was nearly proportional to the length of each chromosome. However, the distribution of discriminating loci between marine and lake ecological forms of Pacific herring was extraordinarily uneven. Most were found on the seventh, twelfth, and twentieth chromosomes (Figure 2).

**Figure 3:**
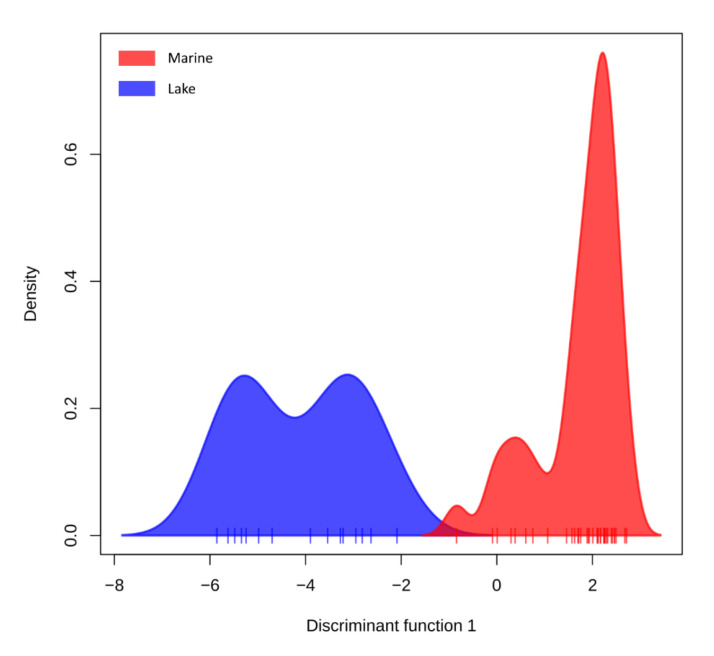
Density plot of Pacific herring specimens along the first discriminant function from Discriminant Analysis of Principal Components (DAPC). Two ecological forms of Pacific herring are shown using different colors. The marine ecological form is in (red), and the lake ecological form (blue).

We also conducted discriminant analyses of the principal component between marine and lake ecological forms of Pacific herring. To explore differences, we combined SNPs datasets from Ainskoe, Nerpiche, and Bolshoy Vilyuy lake populations in one lake group and the remaining in the marine group. As a result, the sample density along the discriminant function separates two ecological groups (Figure 3).

### 3.3. Genomic Divergence Islands between Marine and Lake Ecological Forms of Pacific Herring

SNPs localization in gene bodies was carried out by the intersection of their coordinates with the Atlantic herring reference genome GFF file (Ch_v2.0.2, https://www.ncbi.nlm.nih.gov/assembly/GCF_900700415.2 (accessed on 4 July 2022)). In total, we found SNPs in 10,649 genes of the reference genome of Atlantic herring. Interestingly, those SNP loci, which discriminate marine and lake ecological forms of Pacific herring, were observed only in 253 genes (Table S2). Moreover, 86 discriminating SNP loci are represented in 6 genes—*dnai1*, *LOC105891580 ral guanine nucleotide dissociation stimulator*, *dlg2 channel-associated protein of synapse-110*, *dcc netrin 1 receptor*, *thoc2*, and *sptan1*, which locate in chromosomes 7, 8, 12, and 20.

**Figure 4:**
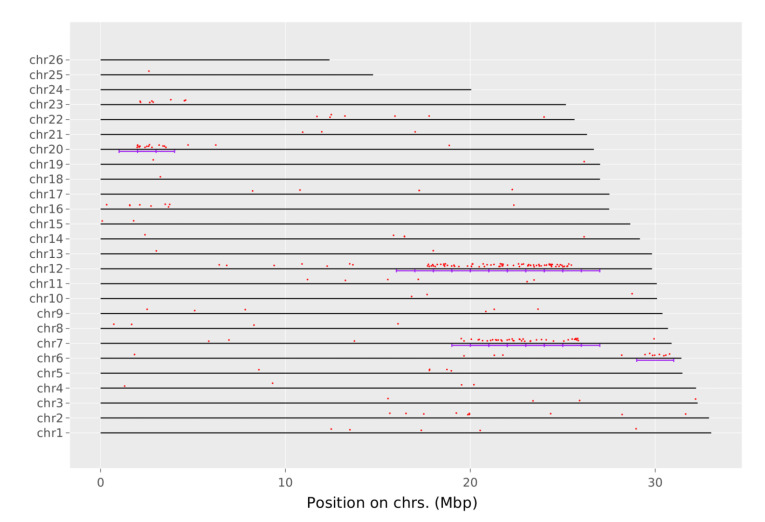
Herring chromosome plot shows the location of discriminating SNP loci (red dots) between marine and lake ecological forms. Genomic divergence islands on chromosomes 6, 7, 12, and 20 are marked by purple lines. The *X*-axis shows the position on the chromosome in Mbp. The *Y*-axis represents chromosomes and their numbers.

Remarkably, chromosomes 7, 8, and 20 contain many discriminating SNP loci (Table S2). The same results were shown by analyzing the list of genes using the ShinyGO web service [46]. Furthermore, a significant number of discriminating SNP loci are localized in chromosomes 6, 7, 12, and 20 and in certain chromosome regions, that were called “genomic islands of divergence” in the previous studies [11,49]. Figure 4 clearly shows how the discriminating SNP loci between the marine and lake ecological forms of Pacific herring are grouped into genomic divergence islands.

Gene ontology (GO) analysis was carried out for the list of 253 genes that contain discriminating SNP loci, using the ShinyGO (v0.76) web service. GO analysis combined 17 of them in neuron development category, which contains 221 gene (Fold Enrichment = 3.2; FDR = 0.033).

Not all discriminating SNP loci between ecological forms of Pacific herring were involved in the analysis since the ddRAD sequencing method does not cover the whole genome and reduces the complexity of the analysis by subsampling only at specific genomic sites defined by restriction enzymes [50].

In total, 133 from 253 genes, which contain discriminating SNP loci between ecological forms of Pacific herring, theare located in genomic divergence islands. Interestingly, these parts of chromosomes 6, 7, 12, and 20 contain 254 genes. Thus, the ddRAD sequencing method used in this study allowed us to determine at least half of them.

Recently, Velotta and colleagues summarized genomic mechanisms of adaptation to different salinity environment conditions in eight different fish species; they described the specific categories of genes that play a crucial role in such adaptation [16]. We found a significant number of these gene categories among our discriminating genes. Strikingly, we speculate that they were possibly involved in freshwater adaptation in Pacific herring. Notably, it has been shown that osmoregulation is influenced by *insulin-like growth factor 1 (IGF-1)*, which receptor and associated binding proteins aid in its transport. This hormone is implicated in the proliferation and differentiation of ionocytes of fish gills [16]. The *insulin-like growth factor 2 mRNA binding protein 1 (igf2bp1)* and *insulin receptor substrate 2b (irs2b)* genes are described in the present study as discriminating between marine and lake ecological forms of Pacific herring. We suppose these genes can be involved in the proliferation of gill ionocytes, which are responsible for osmoregulation [51]. In addition, *fibroblast growth factors (Fgf)* are also represented among discriminating genes. Fibroblasts are implicated in forming connective tissue in animals, which is also necessary to protect body tissues from the changing environment. Moreover, it has been shown that *Fgf* underlies phenotypical adaptations of fish [52]. Transmembrane channel genes are important for freshwater adaptation. Previously, ATPase pump, passive ion cotransporter and transient receptor potential (TRP) channel genes were described as part of molecular pathways related to response to the changing of osmotic conditions [16,53]. In the case of the Pacific herring, a minimum of six genes (*abcc8*, *trpm1a*, *trpc4b*, *cacna1bb*, *sclt1*, and *piezo1*) from this category are revealed as discriminating between marine and lake ecological forms. Moreover, seven genes involved in transmembrane transporter activity (*slc4a1b*, *slc39a10*, *slc15a4*, *slc20a1a*, *slc25a25b*, *slc45a2*, and *slc16a5a*) and three genes involved in calcium homeostasis (*cabp1*, *camk2a*, and *cacna1bb*) were also described. Genome epigenetic modifications and non-coding RNAs play a significant role in the rapid adaptation to changes in the salinity of the environment in fish [15,54,55,56,57]. Table S2 represents several such genes, including *piwil1* (*piwi-like1 RNA-mediated gene*).

The discriminating genes were tested for neutrality using Tajima′s D statistical test. As a result, we obtained weakly positive average Tajima′s D values for both groups of genes — discriminating (0.2044859) and the set of the other genes (0.3041617) with a high degree of significance (*p*-value = 9.042 × 10^−5^) for the 54 Pacific herring specimens. However, Tajima′s D values became sharply negative when we tested only specimens from the lake ecological form. The significantly negative values of Tajima′s D indicate the genes with an excess of low-frequency variation, which is consistent with the fact that the populations might have experienced an expansion after a recent bottleneck, or the genes targeted are under positive selective pressure. Moreover, the difference in the averages of Tajima′s D values for both groups of genes — discriminating (-40.30702) and the whole set of genes (-26.99115) in lake populations have a high degree of significance (*p*-value = 1.185 × 10^−10^). The most logical interpretation of this result is that in addition to the population demographic effect, which can be seen by the Tajima′s D for the whole gene set (-26.99), there is an additional effect of directional selection (-40.3), which is closely related to discriminating genes of the individuals from lake populations of pacific herring.

## 4. Discussion

Our previous studies allowed us to describe the genetic differentiation between the Pacific herring populations in the Russian part of its range using mitochondrial and nuclear genome markers [21,26]. Moreover, the comparative analysis of the genetic differentiation between the combined three lakes on one side and nine marine populations of Pacific herring on the other showed that lake form populations differ from the marine ones [26]. The origin of lake populations across the Russian Far East is still unclear. However, they most likely arose independently due to the significant geographical distance between Sakhalin Island and the Kamchatka Peninsula habitats. Nevertheless, Pacific herring individuals who inhabit fresh-/brackish water in different regions are clustered together based on genetic data [26]. Perhaps, such differences are explained by their ongoing contact with marine individuals, which can spread “freshwater” alleles between lake populations across Russian Far East.

In the present study, even though the relatively small number of gene categories most likely participating in the freshwater adaptation in Pacific herring, we succeeded in showing that the function of most of them are related to osmotic regulation. Moreover, in other teleost species, several of these genes have previously been correlated to the adaptation dynamic toward different salinity conditions [16]. In addition, among discriminative genes between marine and lake forms of Pacific herring, we have found a significant number of regulatory genes, which suggests that rearrangement of regulatory pathways with salinity-related environment changes is a way to quick adaption. Previously, it has been shown on the three-spined stickleback model where at least half of the genes from the genomic divergence islands have regulatory functions [10,11].

Non-coding RNAs are also implicated significantly in the freshwater adaptation of teleost fish by gene expression regulation. Moreover, the differentiation in the microRNA expression occurs in both cases when fish transfer from the marine to the freshwater environment and vice versa—physiological (quick) response or during the time-long period of adaptation (via generations)—evolutionary response. Interestingly, the different microRNAs are expressed in these two types of responses [15].

However, note that discriminative genes between marine and lake ecological forms of Pacific herring are not intersected directly with genes that evolved under positive selection in other fish species, like three-spined stickleback [11]. Indeed, that result is not unexpected. Such overlapping was not observed even between closely related three- and nine-spined (*Pungitius pungitius*) stickleback species [58], which have a possibility for intergeneric hybridization in natural conditions [59,60]. This observation perhaps could be expanded to other types of adaptation among species.

We also compared our discriminating gene list of marine and lake ecological forms of Pacific herring with the same one for Atlantic herring (the Atlantic Ocean and moderate-salted Baltic Sea populations) previously published [17]. Surprisingly, only microRNA silencing modulator*—piwil1* overlapped among the two herring species. Thus, we hypothesize that microRNA-silencing is an essential factor in the process of ecological adaptation of fish species. However, it is not entirely accurate to compare our results with those of Barrio and colleagues since they only analyzed marine specimens which inhabit regions of different salinity [17]. Nevertheless, the gene families and categories associated with freshwater adaptation usually overlap between Pacific herring and other studied species [16]. However, the neuron development GO category has not been described previously as adaptive for changing salinity conditions.

Finally, we suggest that fish species go on their way to achieving their evolutionary goal—an adaptation to new environmental conditions. In addition to the necessary changes that adjust the physiological parameters, such as changes in ion channel activity, several unobvious genetic and phenotypical changes appear during the adaptation to the environment, which differ from species to species. Neuron development category innovations in the lake form of Pacific herring may allow adapting faster to the new ecological niches where this species meets with the new osmotic conditions, predators, changes in feeding, seasonal, sexual, migratory, and other forms of behavior.

## Figures and Tables

**Table 1:** The list of Pacific herring genomic datasets that were used in this study.

Ecological Form	Population Name (Abbreviation)	NCBI Accession Number
Marine (*n* = 39)	Bering Sea, Gulf of Anadyr (Scva)	SRR14076746; SRR14076757; SRR14076758
	Sea of Japan, Aleksandrov Bay (Salex)	SRR14076740; SRR14076741; SRR14076742; SRR14076743; SRR14076744
	Sea of Japan, Amur Bay (Samur)	SRR14076734; SRR14076736; SRR14076737; SRR14076738; SRR14076739
	Kara Sea (Scvk)	SRR14076713; SRR14076724; SRR14076735
	Shelikhov Gulf, Sea of Okhotsk (Seve)	SRR14076729; SRR14076730; SRR14076731; SRR14076732; SRR14076733
	Bering Sea, seaward border of the continental shelf (Sk12)	SRR14076723; SRR14076725; SRR14076726; SRR14076727; SRR14076728
	Bering Sea, Karagin Bay (Skrg)	SRR14076718; SRR14076719; SRR14076720; SRR14076721; SRR14076722
	Kuril Islands, Pacific Ocean (Sukur)	SRR14076712; SRR14076714; SRR14076715; SRR14076716; SRR14076717
	Sea of Okhotsk, Tugur Bay (Sclu7)	SRR14076709; SRR14076710; SRR14076711
Lake (*n* = 15)	Ainskoe Lake, Sakhalin Island (Fain)	SRR14076705; SRR14076706; SRR14076707; SRR14076708; SRR14076756
	Nerpiche Lake, Kamchatka Peninsula (Fnerp)	SRR14076751; SRR14076752; SRR14076753; SRR14076754; SRR14076755
	Bolshoy Vilyuy Lake, Kamchatka Peninsula (Fvil)	SRR14076745; SRR14076747; SRR14076748; SRR14076749; SRR14076750

**Table 2:** Ecological information (average salinity and temperature) in the sampling places across the Pacific Ocean during late spring–early summer.

Ecological Form	Population Name	Abbreviation	Salinity(‰)	Temperature(°C)	Reference
Marine(*n* = 39)	Bering Sea, Gulf of Anadyr	Scva	32.0–34.0	7.0–10.0	[28]
	Sea of Japan, Aleksandrov Bay	Salex	≈29.5	13.0–17.0	[29]
	Sea of Japan, Amur Bay	Samur	23.0–31.0	14.0–22.0	[30]
	Kara Sea	Scvk	20.0–27.0	>10.0–12.0	[31,32]
	Shelikhov Gulf, Sea of Okhotsk	Seve	22.0–33.4	10.0–18.0	[33]
	Bering Sea, seaward border of the continental shelf	Sk12	32.0–34.0	7.0–10.0	[28]
	Bering Sea, Karagin Bay	Skrg	32.0–34.0	7.0–10.0	[28]
	Kuril Islands, Pacific Ocean	Sukur	33.2–33. 4	6.0–12.0	[33]
	Sea of Okhotsk, Tugur Bay	Sclu7	22.0–33.4	10.0–18.0	[33]
Lake (*n* = 15)	Ainskoe Lake, Sakhalin Island	Fain	≈2.5	15.0–20.0	[34]
	Nerpiche Lake, Kamchatka Peninsula	Fnerp	≈4.0	≈10.0	[35,36]
	Bolshoy Vilyuy Lake, Kamchatka Peninsula	Fvil	≈ 1.7	11.0–17.0	[37]

## Data Availability

Double digestion restriction site-associated DNA (ddRAD) sequencing dataset of 54 individuals of Pacific herring is available for download through National Center for Biotechnology Information (NCBI): http://www.ncbi.nlm.nih.gov/, PRJNA717668 (accessed on 13 October 2022).

## Supplementary Materials

The following supporting information can be downloaded at: https://www.mdpi.com/article/10.3390/genes13101856/s1, Table S1: Illumina generated reads and ratio of mapped reads to Atlantic herring (*Clupea harengus*) reference genome sequence (Ch_v2.0.2); Table S2: Genes containing of discriminating SNP loci between marine and lack ecological forms of Pacific herring (*Clupea pallasii*).
